# Diagnosis of parotid gland tumors using a ternary classification model based on ultrasound radiomics

**DOI:** 10.3389/fonc.2025.1485393

**Published:** 2025-03-21

**Authors:** Xiaoling Liu, Weihan Xiao, Chen Yang, Zhihua Wang, Dong Tian, Gang Wang, Xiachuan Qin

**Affiliations:** ^1^ Department of Ultrasound, Beijing Anzhen Nanchong Hospital, Capital Medical University (Nanchong Central Hospital), Nanchong, Sichuan, China; ^2^ School of Medical Imaging, North Sichuan Medical College, Nanchong, China; ^3^ Department of Thoracic Surgery, West China Hospital, Sichuan University, Chengdu, China; ^4^ Department of Ultrasound, Shaoyang Central Hospital, Shaoyang, China; ^5^ Department of Ultrasound, Chengdu Second People’s Hospital, Chengdu, China

**Keywords:** parotid gland tumors, pleomorphic adenoma, Warthin’s tumor, ultrasound radiomics, classification diagnosis

## Abstract

**Objective:**

This study aimed to evaluate the diagnostic value of two-step ultrasound radiomics models in distinguishing parotid malignancies from pleomorphic adenomas (PAs) and Warthin’s tumors (WTs).

**Methods:**

A retrospective analysis was conducted on patients who underwent parotidectomy at our institution between January 2015 and December 2022. Radiomics features were extracted from two-dimensional (2D) ultrasound images using 3D Slicer. Feature selection was performed using the Mann–Whitney *U* test and seven additional selection methods. Two-step LASSO-BNB and voting ensemble learning modeling algorithm with recursive feature elimination feature selection method (RFE-Voting) models were then applied for classification. Model performance was assessed using the area under the receiver operating characteristic curve (AUC), and internal validation was conducted through fivefold cross-validation.

**Results:**

A total of 336 patients were included in the study, comprising 73 with malignant tumors and 263 with benign lesions (118 WT and 145 PA). The LASSO-NB model demonstrated excellent performance in distinguishing between benign and malignant parotid lesions, achieving an AUC of 0.910 (95% CI, 0.907–0.914), with an accuracy of 86.8%, sensitivity of 92.5%, and specificity of 66.7%, significantly outperforming experienced sonographers (accuracy of 61.90%). The RFE-Voting model also showed outstanding performance in differentiating PA from WT, with an AUC of 0.962 (95% CI, 0.959–0.963), accuracy of 83.0%, sensitivity of 84.0%, and specificity of 92.1%, exceeding the diagnostic capability of experienced sonographers (accuracy of 65.39%).

**Conclusion:**

The two-step LASSO-BNB and RFE-Voting models based on ultrasound imaging performed well in distinguishing glandular malignant tumors from PA and WT and have good predictive capabilities, which can provide more useful information for non-invasive differentiation of parotid gland tumors before surgery.

## Introduction

Parotid gland tumors are mostly benign, of which pleomorphic adenoma (PA) and Warthin’s tumor (WT) account for the vast majority, accounting for 85.7% (1), and the risk of malignant tumors (MTs) is 7%–15%. Therefore, the identification of PA and WT in benign parotid tumors, as well as their identification in MTs, has become the main content of daily ultrasound diagnosis of parotid tumors. Accurately distinguishing between benign and malignant tumors, as well as differentiating between PA and WT, can help radiologists solve over 90% of parotid gland tumor diagnosis problems such as the physiological function of the parotid gland and the anatomical structure of the parotid gland area are complex, the capsule of the parotid gland tumor is incomplete, the tissue cutting biopsy of the thick needle is prone to tumor implantation and metastasis, and the scar formed after the biopsy easily increases the risk of intraoperative facial nerve injury. Therefore, the parotid gland tumor is generally not cut before surgery. Biopsy for preoperative diagnosis depends on patient signs and imaging findings. The parotid glands may be quickly and inexpensively examined using sonography; in ultrasound, the pleomorphic adenoma displays smooth margins, often with lobules and either homogeneous or heterogenic parenchyma. Doppler sonography typically shows little vascularization. Calcifications as well as necrosis tend to appear as the tumor becomes larger (2). However, given the overlap and atypicality of imaging characteristics, the accuracy of using ultrasound alone to diagnose PA is approximately 64% and 82% for Warthin’s tumor. This presents a significant challenge in distinguishing between benign and malignant parotid tumors, as well as their common subtypes, based solely on clinical and imaging evaluations (3).

Radiomics is an emerging technology that is defined as transforming medical images into high-throughput features to quantitatively evaluate tumor phenotypes (4, 5). High-throughput features that cannot be directly observed by the naked eye can be quantitatively extracted from single or multiple medical images and then applied to machine learning methods to construct classification or prediction models. This method can more objectively evaluate tumor status and distinguish malignant from benign tumors or nodules (6, 7). Therefore, we attempted to use a ternary classification method (8) based on ultrasound radiomics analysis to improve the diagnostic confidence of radiologists in the diagnosis of parotid nodules, which is helpful for physicians to formulate more accurate treatment plans before surgery, shorten the operation time of patients, predict the outcome of the disease, and provide a more accurate and non-invasive preoperative diagnosis for the clinic.

## Method

### Study design and population

The study was approved by the Institutional Ethics Review Committee, which waived the requirement for informed consent. It involved only anonymous imaging datasets, and no individual patient data or human tissue samples were collected.

A total of 336 patients who underwent parotid gland mastectomy in Nanchong Central Hospital from January 2015 to December 2022 were retrospectively analyzed. The clinicopathological and ultrasound data of the patients were retrospectively analyzed. The inclusion criteria were as follows: 1) patients underwent surgery and pathological results, 2) underwent routine ultrasound examination within 1 week before surgery, and 3) had complete imaging data. The exclusion criteria were as follows: 1) patients receiving neoadjuvant therapy (chemotherapy, immunotherapy, or radiotherapy) and 2) were uncooperative during an ultrasound examination or had an image quality that was poor and not suitable for analysis.

The ultrasound (US) instruments employed to acquire the images used in this study included Mindray Resona 7 (Shenzhen Mindray Bio-Medical Electronics Co., Ltd., Shenzhen, China), Esaote MyLab (Esaote, Genoa, Italy), and linear array probe frequency (8–12 MHz). Considering that the corresponding diagnostic results of transverse and longitudinal assessments may overlap and that the longitudinal section is less disturbed, our study selected the longitudinal section of the tumor.

### Imaging segmentation and feature extraction

All images were anonymized and measured using the 3D Slicer software (version 4.10.2, https://www.slicer.org). The measurement was completed by two physicians with more than 5 years of experience in ultrasound diagnosis who were blinded to the pathological results.

The feature extraction was implemented using the open-source python package “Pyradiomics V3.0.1” (http://www.radiomics.io/pyradiomics.html). Then, a total of 474 radiomics features were extracted.

We standardized the gray value of all ultrasound images to ensure that the gray value range was consistent and did not need resampling because ultrasound is a two-dimensional (2D) image and does not contain three-dimensional (3D) spatial information. After we ensured that the mask matched the image, we used the same radiomics feature extractor for feature extraction to ensure that the extracted radiomics feature extraction was consistent and reliable.

### Feature filtering and model building

Similar methods were used in other studies by our team (9). All clinical features (15) and radiomics features (474)were selected using the Mann–Whitney *U* test after z-score normalization, and then seven feature selection methods (least absolute shrinkage and selection operator, analysis of variance, mutual information, recursive feature elimination, forward selection, random forest, and logistic regression) were used for further selection. The adjustment parameter (λ) in the LASSO model was selected using fivefold cross-validation, and the grid search method was used to adjust the parameters to obtain the LASSO coefficient spectrum of radiomics and clinical features. Subsequently, 11 modeling algorithms were used to model the features selected by each feature screening method. A total of 77 models were established, and heatmaps showing the area under the curve of each model were obtained.

#### Filter methods

Mutual information (MI) is a measure of the degree of interdependence between two random variables. It is based on the concept of entropy in information theory, indicating how much uncertainty of one variable can be reduced by another variable. MI is not limited to linear relationships; it can capture any type of statistical dependence, including non-linear relationships. This method is simple and efficient and can quickly screen out features related to disease diagnosis and reduce the complexity of the model.

ANOVA is mainly used to compare the mean values between three or more groups to determine that there is a significant difference between the mean values of at least one group and other groups. Because our clinical data and omics data are mostly continuous variables, this method is selected. Through ANOVA, the features that have a significant impact on disease diagnosis can be screened out, and the accuracy and generalization ability of the model can be improved.

#### Wrapper methods

Recursive feature elimination (RFE): By recursively training the model, the least important features are removed until a predetermined number of features are reached. RFE can dynamically select features based on the performance of the model, ensuring that the final selected features significantly improve the predictive ability of the model.

Forward search (FS) is suitable for the case where the number of features is large and the computing resources are limited. This method starts with a basic model without any features and then adds a feature in turn until there is no more improvement. It provides an effective method to reduce the feature dimension while maintaining the predictive performance of the model as much as possible.

#### Embedded methods

LASSO belongs to the method of L1 regularization (LASSO Regression): by adding L1 norm terms to the loss function, the weights of certain features become zero, thereby achieving feature selection. L1 regularization can not only reduce the number of features but also improve the sparsity and interpretability of the model.

Feature importance from tree-based models: Feature importance scores from random forest tree models are used to select important features. The tree model can automatically evaluate the importance of features and select the features that contribute the most to disease diagnosis.

### Model evaluation

The fivefold cross-validation method was used to test the best model performance for differentiating benign from MT and differentiating PA from WT; the area under the curve (AUC), sensitivity, specificity, positive predictive value (PPV), negative predictive value (NPV), and accuracy of the two models were obtained.

The receiver operating characteristic (ROC) curve and decision curve analysis (DCA) were used to evaluate the optimal model for differentiating benign tumors from MT, as well as PA from WT.

### Comparison of models by experienced doctors

Two expert radiologists (with 13 and 20 years of experience in ultrasound diagnosis of parotid gland diseases) studied the images. Neither of the radiologists knew the patients’ clinical features and pathological results. First, 263 cases of benign and 73 cases of malignant lesions were differentiated among all cases, and the benign and malignant cases were determined by ultrasound images. Furthermore, 118 cases of WT and 145 cases of PA in benign lesions were differentiated and diagnosed. If there was any disagreement, both parties reached a consensus through discussion. The results were compared with those of the best model. In case of differences, a consensus was reached through mutual discussion. The results were compared with those of the best model.

### Statistical analysis

Statistical analysis was performed using SPSS 25.0 (IBM, Armonk, NY, USA). First, the Kolmogorov–Smirnov test was used to evaluate whether the count data were normally distributed. The independent samples *t*-test was used when the normal distribution was met, and the Mann–Whitney *U* test was used when the normal distribution was not met. Continuous variables were described as mean ± SD, and enumeration data were compared among groups using χ^2^ test or Fisher’s exact probability. ROC curve was used to evaluate the accuracy, sensitivity, specificity, and AUC value of the two best models. *p* < 0.05 was considered to be statistically significant.

## Results

### Clinical and ultrasound image characteristics of patients

In this study, there were 336 patients with parotid tumors: 263 benign and 73 malignant. In the benign cases, there were 118 cases of WT and 145 cases of PA. The clinical features and ultrasonographic features of benign and malignant parotid tumors are shown in **Table 1**. The clinical characteristics and ultrasound manifestations of benign parotid gland tumors—pleomorphic adenoma and Warthin’s tumor—are shown in **Table 2**.

**Table 1:** Baseline clinical characteristics of all patients.

Characteristics	Benign (N = 263)	Malignant (N = 73)	All patients (N = 336)	t/χ^2^	*p*
Sex				12.566	0.000
Male	172 (65.4%)	31 (42.5%)	203 (60.4%)		
Female	91 (34.6%)	42 (57.5%)	133 (39.6%)		
Age (years)	52.43 ± 14.11	53.30 ± 17.64	52.62 ± 14.92	0.440	0.660
Site				1.938	0.164
Left	138 (52.5%)	45 (61.6%)	183 (54.5%)		
Right	125 (47.5%)	28 (38.4%)	153 (45.5%)		
Long diameter (mm)	26.59 ± 10.42	29.38 ± 11.73	27.20 ± 10.76	1.968	0.050
Short diameter (mm)	17.05 ± 7.60	20.10 ± 7.94	17.71 ± 7.76	3.004	0.003
Multiple				2.21	0.137
Negative	243 (92.4%)	71 (97.3%)	314 (93.5%)		
Positive	20 (7.6%)	2 (2.7%)	22 (6.5%)		
Shape				36.490	0.000
Irregular	39 (14.8%)	35 (47.9%)	74 (22.0%)		
Regular	224 (85.2%)	38 (52.1%)	262 (78.0%)		
Boundary				6.796	0.009
Unclear	12 (4.6%)	51 (69.9%)	63 (18.8%)		
Clear	251 (95.4%)	22 (30.1%)	273 (81.3%)		
Echo				1.120	0.290
Low-echo	259 (98.5%)	73 (100.0%)	332 (98.8%)		
Iso-echo	4 (1.5%)	0 (0.0%)	4 (1.2%)		
Anecho	0 (0.0%)	0 (0.0%)	0 (0.0%)		
Homogeneity				24.424	0.000
Heterogeneous	131 (49.8%)	60 (82.2%)	191 (56.8%)		
Homogeneous	132 (50.2%)	13 (17.8%)	145 (43.2%)		
Liquidation				8.371	0.004
Negative	144 (54.8%)	26 (35.6%)	170 (50.6%)		
Positive	119 (45.2%)	47 (64.4%)	166 (49.4%)		
Grid				21.944	0.000
Negative	199 (75.7%)	73 (100.0%)	272 (81.0%)		
Positive	64 (24.3%)	0 (0.0%)	64 (19.0%)		
Calcification				34.576	0.000
Negative	251 (95.4%)	53 (72.6%)	304 (90.5%)		
Positive	12 (4.6%)	20 (27.4%)	32 (9.5%)		
Enhancement of behind echo				36.449	0.000
Negative	104 (39.5%)	58 (79.5%)	162 (48.2%)		
Positive	159 (60.5%)	15 (20.5%)	174 (51.8%)		
Alder				7.862	0.049
Level 0	21 (8.0%)	9 (12.3%)	30 (8.9%)		
Level 1	110 (41.8%)	24 (32.9%)	134 (39.9%)		
Level 2	84 (31.9%)	33 (45.2%)	117 (34.8%)		
Level 3	48 (18.3%)	7 (9.6%)	55 (16.4%)		

**Table 2:** Baseline clinical characteristics of all patients with benign parotid gland.

Characteristics	Warthin (N = 118)	Pleomorphic adenoma (N = 145)	All patients (N = 263)	t/χ^2^	*p*
Sex				97.206	0.000
Male	115 (97.5%)	57 (39.3%)	172 (65.4%)		
Female	3 (2.5%)	88 (60.7%)	91 (34.6%)		
Age (years)	58.66 ± 9.17	47.36 ± 15.36	52.43 ± 14.11	7.035	0.000
Site				0.268	0.621
Left	64 (54.2%)	74 (51.0%)	138 (52.5%)		
Right	54 (45.8%)	71 (49.0%)	125 (47.5%)		
Long diameter (mm)	28.47 ± 11.47	25.06 ± 9.24	26.59 ± 10.42	2.670	0.008
Short diameter (mm)	17.75 ± 9.18	16.48 ± 5.98	17.05 ± 7.60	1.351	0.178
Multiple				2.004	0.157
Negative	106 (89.8%)	137 (94.5%)	243 (92.4%)		
Positive	12 (10.2%)	8 (5.5%)	20 (7.6%)		
Shape				3.679	0.055
Irregular	12 (10.2%)	27 (18.6%)	39 (14.8%)		
Regular	106 (89.8%)	118 (81.4%)	224 (85.2%)		
Boundary				0.134	0.714
Unclear	6 (5.1%)	6 (4.1%)	12 (4.6%)		
Clear	112 (94.9%)	139 (95.9%)	251 (95.4%)		
Echo				3.293	0.070
Low-echo	118 (100%)	141 (97.2%)	259 (98.5%)		
Iso-echo	0 (0.0%)	4 (2.8%)	4 (1.5%)		
Anecho	0 (0.0%)	0 (0.0%)	0 (0.0%)		
Homogeneity				4.159	0.041
Heterogeneous	67 (56.8%)	64 (44.1%)	131 (49.8%)		
Homogeneous	51 (43.2%)	81 (55.9%)	132 (50.2%)		
Liquidation				1.318	0.251
Negative	60 (50.8%)	84 (57.9%)	144 (54.8%)		
Positive	58 (49.2%)	61 (42.1%)	119 (45.2%)		
Grid				53.372	0.000
Negative	64 (54.2%)	135 (93.1%)	199 (75.7%)		
Positive	54 (45.8%)	10 (6.9%)	64 (24.3%)		
Calcification				0.676	0.411
Negative	114 (96.6%)	137 (94.5%)	251 (95.4%)		
Positive	4 (3.4%)	8 (5.5%)	12 (4.6%)		
Enhancement of behind echo				1.210	0.271
Negative	51 (43.2%)	53 (36.6%)	104 (39.5%)		
Positive	67 (56.8%)	92 (63.4%)	159 (60.5%)		
Alder				70.015	0.000
Level 0	1 (0.8%)	20 (13.8%)	21 (8.0%)		
Level 1	25 (21.2%)	85 (58.6%)	110 (41.8%)		
Level 2	55 (46.6%)	29 (20.0%)	84 (31.9%)		
Level 3	37 (31.4%)	11 (7.6%)	48 (18.3%)		

### Model based on radiomics combined with clinical features

A total of 474 radiomics features were extracted as follows: a) Shape 2D-based features (n = 9), b) first-order statistical features (n = 18), c) gray-level co-occurrence matrix (GLCM)-based features (n = 24), d) gray-level dependence matrix (GLDM)-based features (n = 14), e) gray-level run-length matrix (GLRLM)-based features (n = 16), f) gray-level size zone matrix (GLSZM)-based features (n = 16), g) neighboring gray-tone difference matrix (NGTDM)-based features (n = 5), and (h) transform-filtered features (including wavelet) (n = 372).

**Figure 1:**
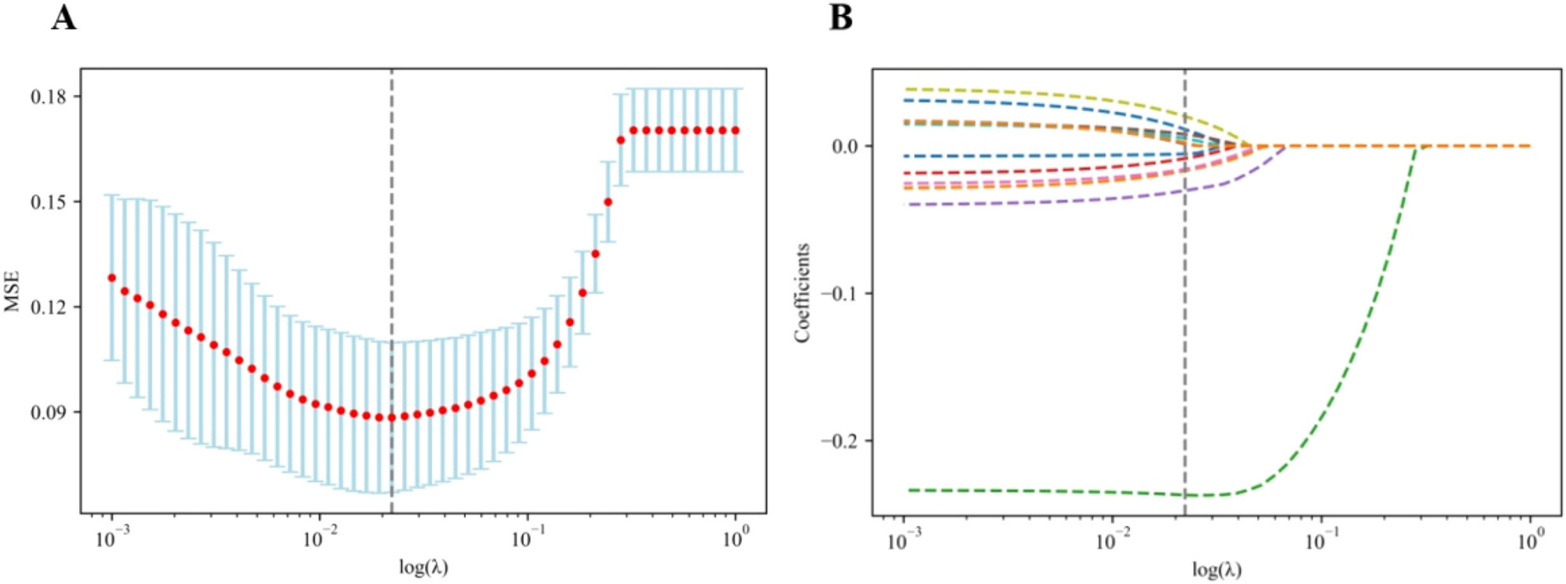
Flowchart of radiomics and clinical feature selection. **(A)** Selection of the tuning parameter (λ) in the LASSO model via the fivefold cross-validation. MSE on each fold from the LASSO regression cross-validation procedure was plotted as a function of log (λ). The optimal λ value of 0.019 was selected. **(B)** LASSO coefficient profiles of the 474 radiomics features and 15 clinical features. A vertical line was drawn at the value identified via the fivefold cross-validation, at which the optimal λ resulted in 12 non-zero coefficients. LASSO, least absolute shrinkage and selection operator.

The optimal λ value of the log (λ) function of MSE was 0.019 by LASSO regression cross-validation, and the LASSO coefficient spectrum was obtained, as shown in **Figure 1**.

**Figure 2:**
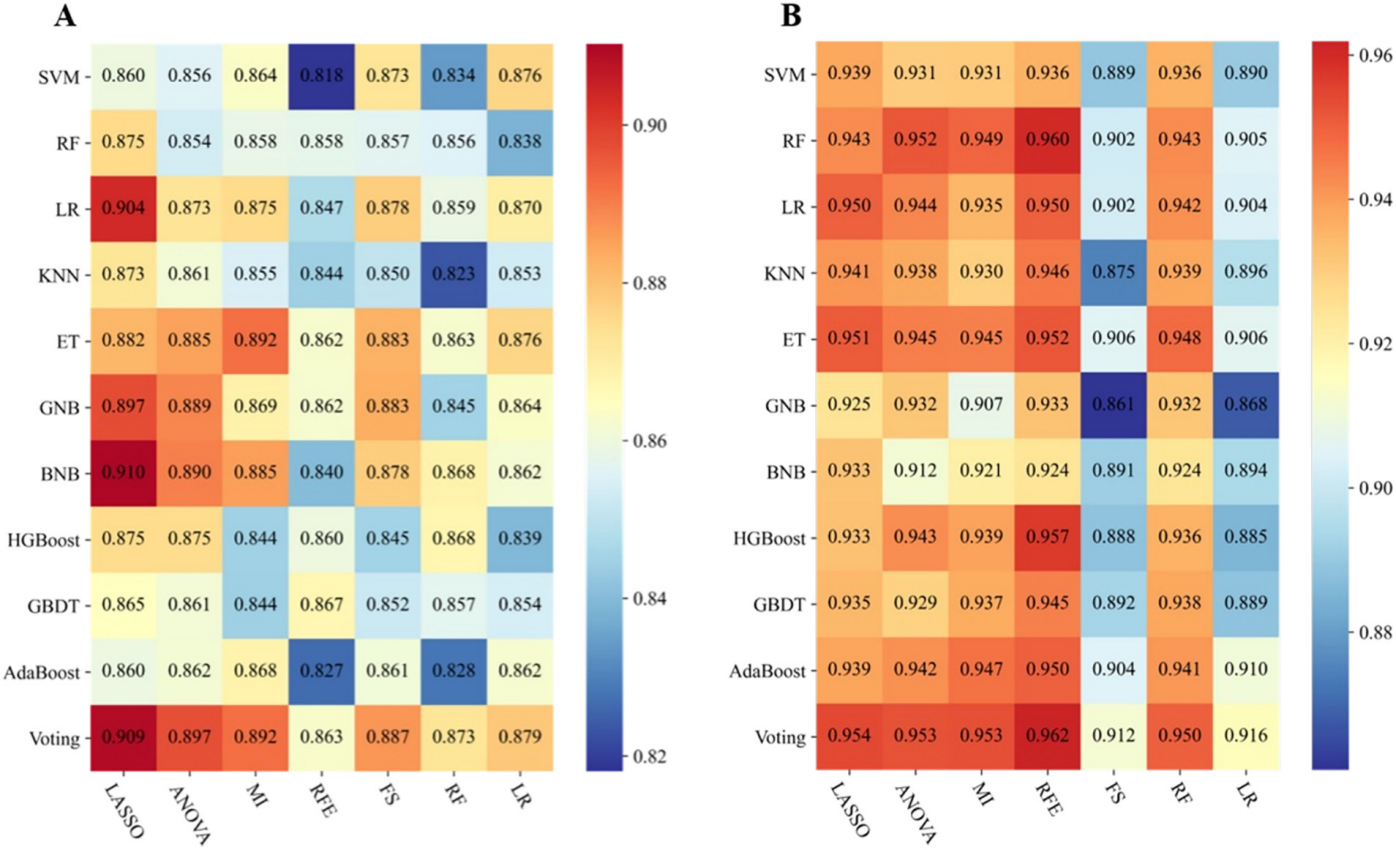
Performance of 77 models incorporating radiomics and clinical features via the fivefold cross-validation. **(A)** Performance of benign and malignant models. **(B)** Performance of Warthin and pleomorphic adenoma models. The heatmaps show the area under the curve of each modeling algorithm (columns) with each feature selection method (rows). SVM, support vector machine; RF, random forest; LR, logistic regression; KNN, k-nearest neighbors; ET, extremely randomized trees; GNB, Gaussian naive Bayes; BNB, Bernoulli naive Bayes; HGBoost, histogram-based gradient boosting; GBDT, gradient boosted decision trees; AdaBoost, adaptive boosting; Voting, voting classifier; LASSO, least absolute shrinkage and selection operator; ANOVA, analysis of variance; MI, mutual information; RFE, recursive feature elimination; FS, forward selection.

Based on 77 models integrating radiomics and clinical features, the heatmaps of the area under the curve (as shown in **Figure 2**) showed that the LASSO-BNB model was superior to other models in differentiating benign from malignant parotid lesions (AUC 0.910). Voting ensemble learning modeling algorithm with recursive feature elimination feature selection method (RFE-Voting) showed the best performance in the differential diagnosis of benign parotid PA and WT (AUC 0.962). The LASSO-BNB and RFE-Voting models were modeled using 12 and 13 features, respectively. The detailed features are shown in **Supplementary Tables 1**, **2**.

### Performance of the best model evaluation of model

**Figure 3:**
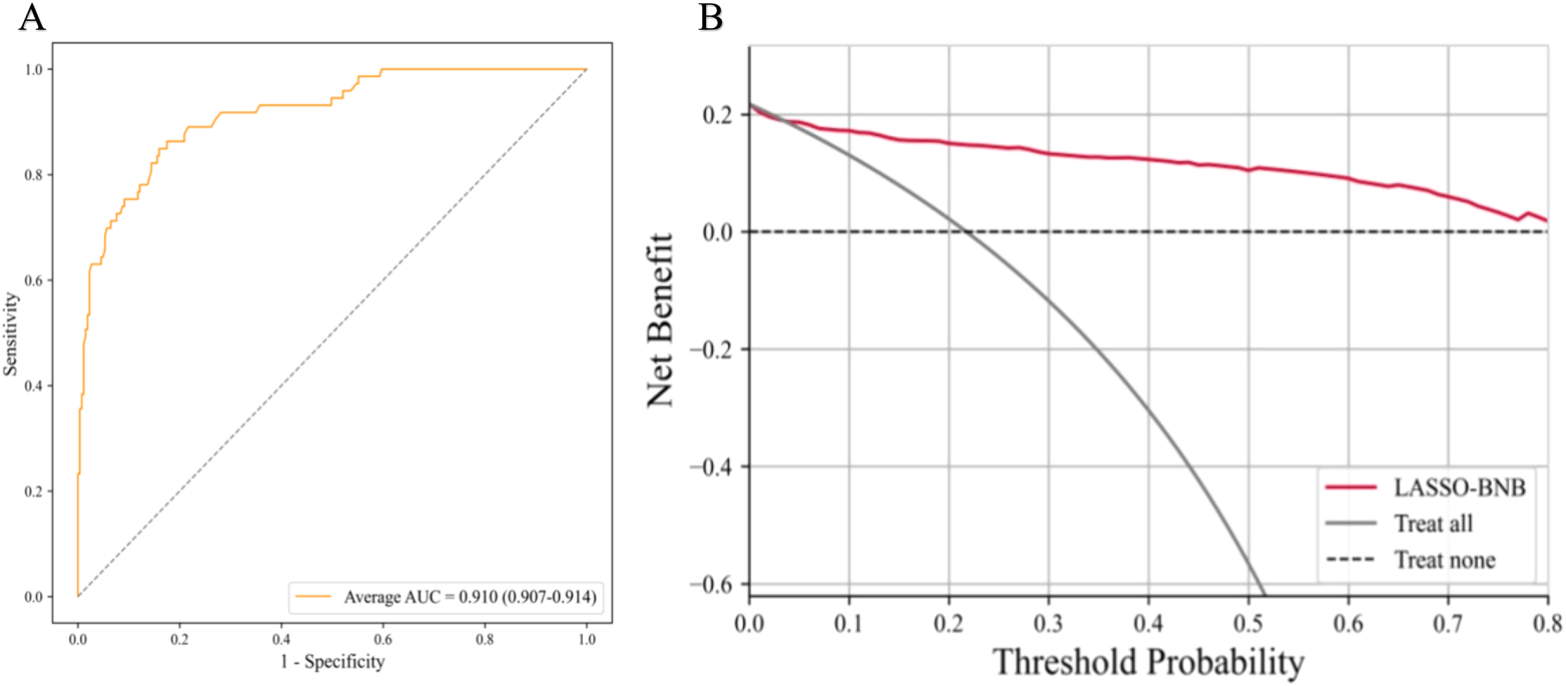
The best model for distinguishing benign and malignant parotid glands is LASSO-BNB. **(A)** The ROC analysis of radiomics and clinical model via the fivefold cross-validation. **(B)** The decision curve analysis of the best performance model. LASSO-BNB, Bernoulli naive Bayes modeling algorithm with least absolute shrinkage and selection operator feature selection method; ROC, receiver operating characteristic curve.

**Figure 4:**
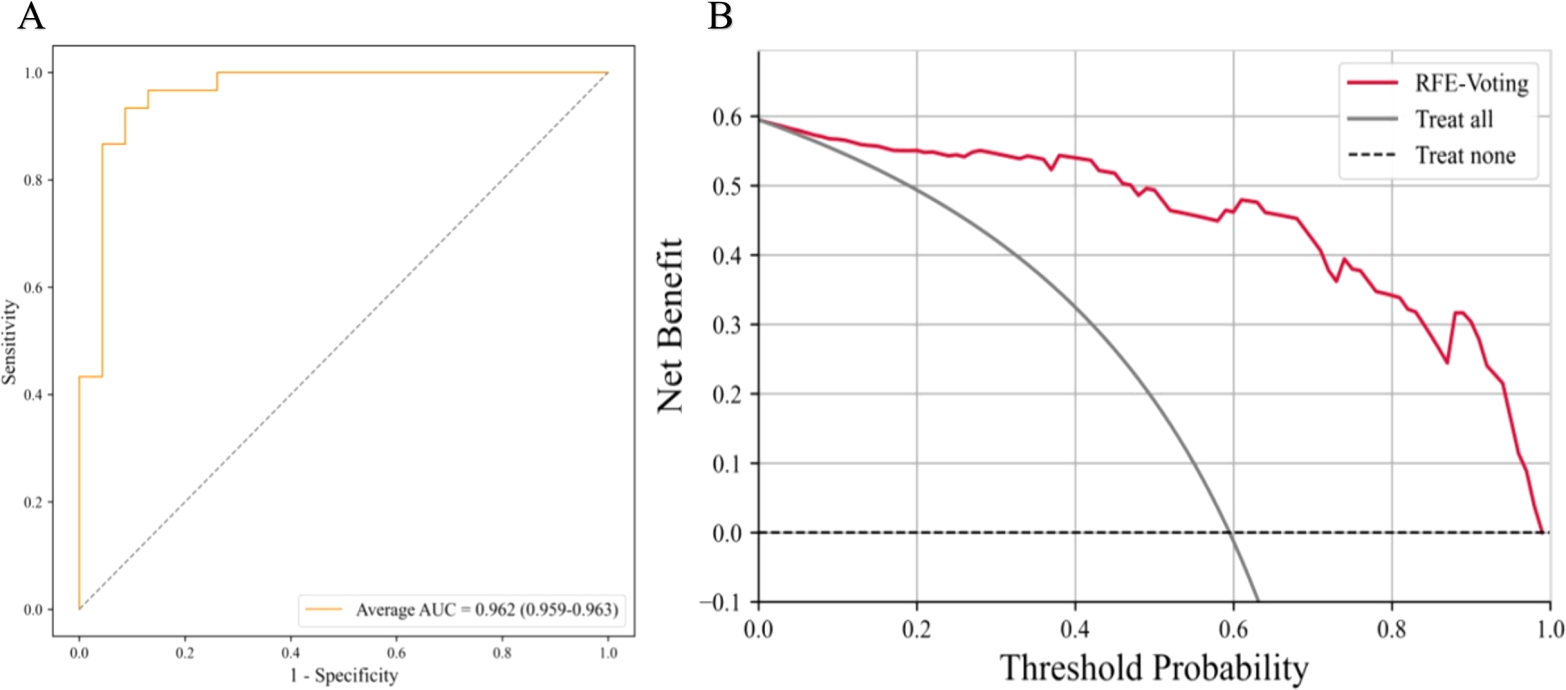
The best model for distinguishing benign parotid glands is RFE-Voting. **(A)** The ROC analysis of radiomics and clinical model via the fivefold cross-validation. **(B)** The decision curve analysis of the best performance model. RFE-Voting, voting ensemble learning modeling algorithm with recursive feature elimination feature selection method. The voting ensemble learning model consists of RFE-RF, RFE-ET, and RFE-HGBoost. RFE-RF, random forest modeling algorithm with recursive feature elimination feature selection method; RFE-ET, extremely randomized trees modeling algorithm with recursive feature elimination feature selection method; RFE-HGBoost, histogram-based gradient boosting modeling algorithm with recursive feature elimination feature selection method; ROC, receiver operating characteristic curve.

The AUC of LASSO-BNB was 0.910 (CI 0.907–0.914) as shown in **Figure 3A**, sensitivity was 92.5%, specificity was 66.7%, PPV was 90.4%, NPV was 71.4%, and accuracy was 86.8%. The AUC of RFE-Voting was 0.962 (CI 0.959–0.963) as shown in **Figure 4A**, sensitivity was 84.0%, specificity was 82.1%, PPV was 80.8%, NPV was 85.2%, and accuracy was 83.0%, as shown in **Table 3**.

**Table 3:** Performance of two best models.

Model	AUC (95% CI)	SEN	SPE	PPV	NPV	ACC	F1 score
**LASSO-BNB**	0.910 (0.907–0.914)	92.5%	66.7%	90.4%	71.4%	86.8%	71.0%
**RFE-Voting**	0.962 (0.959–0.963)	84.0%	82.1%	80.8%	85.2%	83.0%	88.5%

LASSO-BNB, Bernoulli naive Bayes modeling algorithm with least absolute shrinkage and selection operator feature selection method to identify benign or malignant parotid gland; RFE-Voting, voting ensemble learning modeling algorithm with recursive feature elimination feature selection method to identify benign parotid gland; CI, confidence interval; SEN, sensitivity; SPE, specificity; ACC, accuracy; PPV, positive predictive value; NPV, negative predictive value.

The ROC curve based on the best model for distinguishing benign and malignant parotid glands (LASSO-BNB model) is shown in **Figure 3A**. The ROC curve based on the best model for distinguishing benign parotid glands (RFE-Voting model) is shown in **Figure 4A**. The decision curve for evaluating the model is shown in **Figure 3B** and **Figure 4B**.

The accuracy (ACC) of experienced radiologists for distinguishing benign and malignant parotid glands was 61.9%, and for the diagnosis of PA and WT, it was 65.4%.

## Discussion

The tissue composition of parotid gland tumors is complex and lacks clinical symptoms and indicators. Imaging findings can provide some value (10–13), but in the actual operation process, it is susceptible to the subjective judgment and experience of doctors, thus affecting the diagnostic results. As the preferred examination method for parotid tumors, ultrasonography is widely used in the diagnosis of parotid diseases. With the development of some new technologies such as elastography and contrast-enhanced ultrasound, some useful information has been provided for the diagnosis of parotid tumors from the aspects of morphology and blood perfusion (14–17). However, the overlap of ultrasonographic features of benign and malignant parotid tumors and different pathological types of benign lesions leads to some controversy in its clinical application value (18). Ultrasound radiomics features reflect the heterogeneity of the tumor by reflecting the texture information of the tumor, which makes it possible to transform traditional images into image analysis that can mine high-dimensional data information. It can better quantify the lesion features that cannot be distinguished by the naked eye (19–21) and also reduces the subjectivity brought by the diagnostic doctor, which also provides ideas for the differential diagnosis of parotid tumors by ultrasonic image texture analysis.

The proportion of parotid malignant tumors is relatively small, and half of the lesions have no obvious clinical manifestations. Preoperative fine-needle aspiration cytology is considered to be a reliable method, but it is an invasive operation, and the number of cells taken out is limited, so it is not suitable for deep tumors. However, the malignant transformation rate of PA as a benign tumor is 2%–25%, and the recurrence rate after simple lesion resection is 15% (22). WT rarely has malignant transformation and recurrence. Clinically, conservative treatment or simple lesion enucleation can be used. Different tumors have different surgical methods and treatment methods. Therefore, preoperative non-invasive qualitative diagnosis is very important.

According to the literature, there is no report on the simultaneous identification of benign and malignant parotid tumors and PA and WT by ultrasound radiomics. In our study, we combined 474 radiomics features and 15 clinical features extracted from gray-scale ultrasound images and established 77 models after further screening. We observed and compared the diagnostic efficacy of each model. We obtained the best model LASSO-BNB for differentiating benign from malignant tumors. The AUC of the model reached 0.910, and the accuracy was 92.5%, which was much higher than that of clinical ultrasound physician diagnosis and previous conventional ultrasonographic feature analysis. The best model for differentiating benign PA from WT is RFE-Voting. The AUC of this model reached 0.962, and its accuracy was 83.0%. It shows that the radiomics analysis of gray-scale ultrasound images shows sufficient prediction efficiency in differential diagnosis, which provides more confidence for non-invasive diagnosis of parotid tumors before clinical surgery. Compared with the logistic regression conducted by other research (23), our LASSO-BNB model performance is basically the same (AUC: 0.910 vs. 0.914).

The present study has several limitations that require further consideration. First, this was a single-center study with a small sample size, so more center data should be collected in subsequent studies. Second, we only studied the two-dimensional ultrasound of the parotid gland. The multimodal analysis that should be included in subsequent studies may further improve the accuracy.

The dependence on the operator is inevitable, so all our model results were obtained using the method of fivefold cross-validation to find the average value, and the influence of random number seeds on the performance results of the model was reduced as much as possible. This method may be superior to the simple division of the training set and test set.

## Conclusion

This two-step integrated model of ultrasound radiomics and clinical features has achieved outstanding performance in the three-category diagnosis of MT, PA, and WT. It is superior to skilled ultrasound diagnosticians and provides an effective diagnostic reference for clinical routine parotid tumor identification. This method may become a new non-invasive preoperative evaluation method for clinical application.

## Data Availability

The original contributions presented in the study are included in the article/**Supplementary Material**. Further inquiries can be directed to the corresponding authors.

## Glossary

MSE: mean square error
MT: malignant tumor
PA: pleomorphic adenoma
WT: Warthin’s tumor
ROC: receiver operating characteristic
DCA: decision curve analysis
AUC: area under the curve
SVM: support vector machine
RF: random forest
LR: logistic regression
KNN: k-nearest neighbors
ET: extremely randomized trees
GNB: Gaussian naive Bayes
BNB: Bernoulli naive Bayes
HGBoost: histogram-based gradient boosting
GBDT: gradient boosted decision trees
AdaBoost: adaptive boosting
Voting: voting classifier
LASSO: least absolute shrinkage and selection operator
ANOVA: analysis of variance
MI: mutual information
RFE: recursive feature elimination
FS: forward selection
LASSO-BNB: Bernoulli naive Bayes modeling algorithm with least absolute shrinkage and selection operator feature selection method
RFE-Voting: voting ensemble learning modeling algorithm with recursive feature elimination feature selection method
RFE-RF: random forest modeling algorithm with recursive feature elimination feature selection method
RFE-ET: extremely randomized trees modeling algorithm with recursive feature elimination feature selection method
RFE-HGBoost: histogram-based gradient boosting modeling algorithm with recursive feature elimination feature selection method
CI: confidence interval
PPV: positive predictive value
NPV: negative predictive value
