# Supplementary material for: Diagnosis of parotid gland tumors using a ternary classification model based on ultrasound radiomics
Source: Front Oncol. 2025 Mar 21;15:1485393. doi: 10.3389/fonc.2025.1485393 (PMC11968691; doi:10.3389/fonc.2025.1485393)
Supplement: Supplementary file 1 [file DataSheet1.docx]

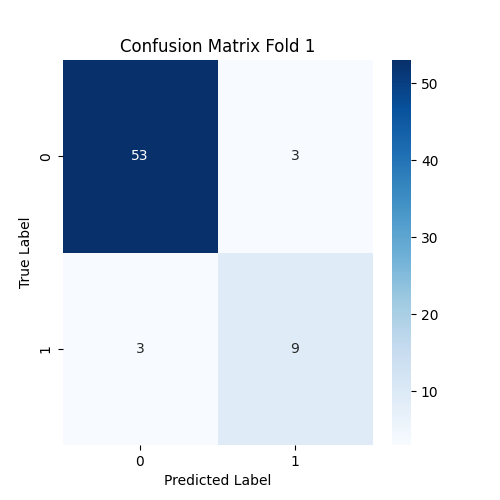

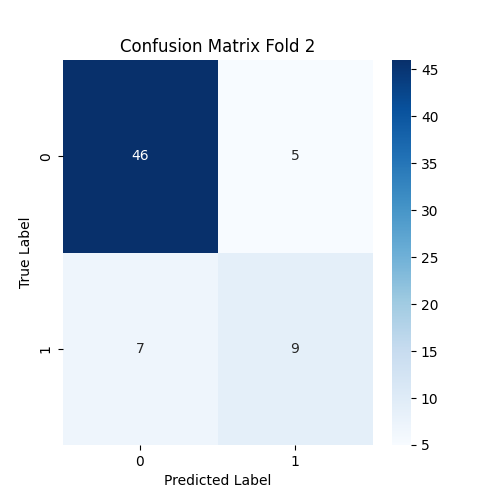

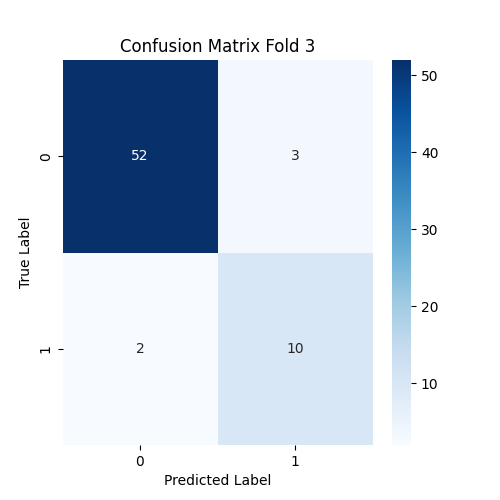

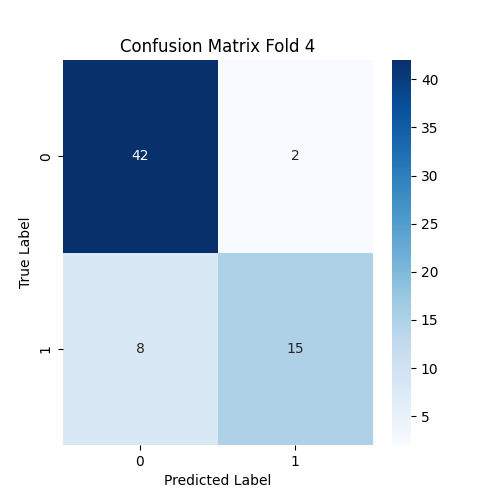

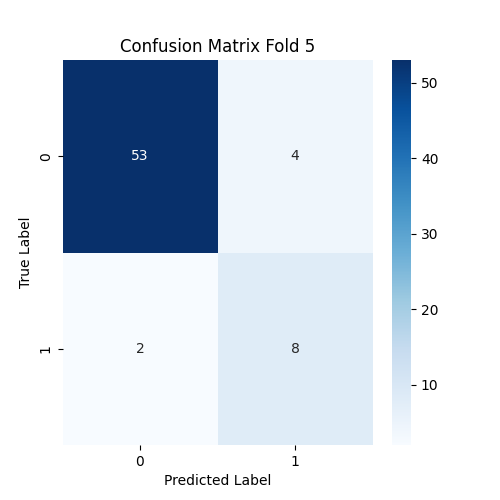


**Supplement Figure 1: Confusion matrix diagram of five-fold cross-validation of LASSO-BNB**

**
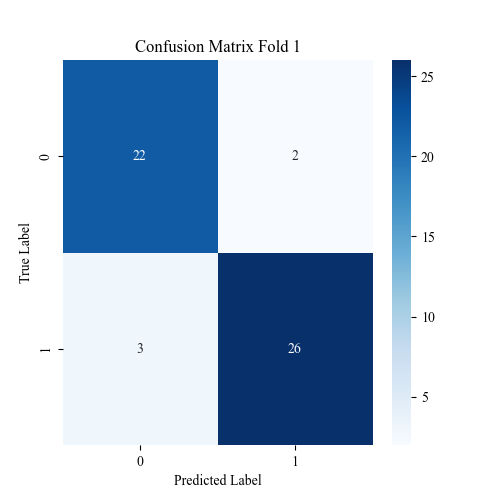

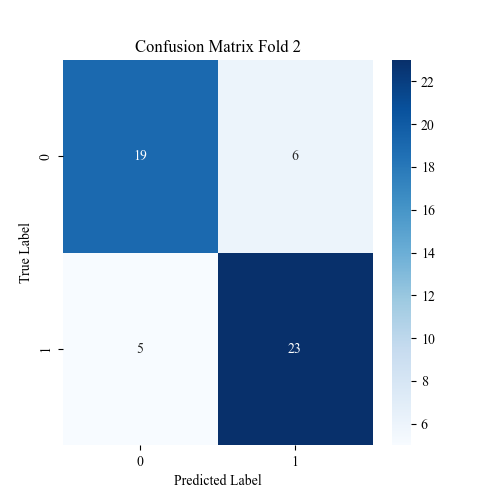

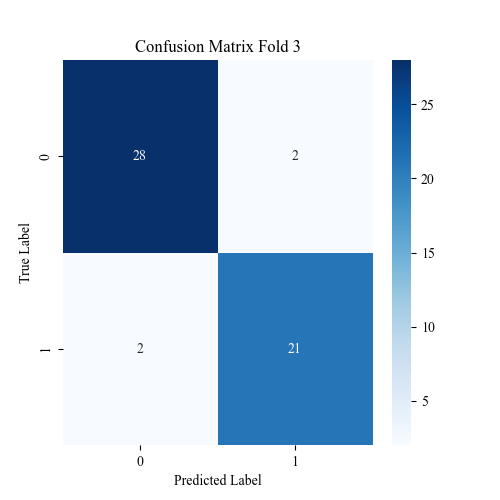

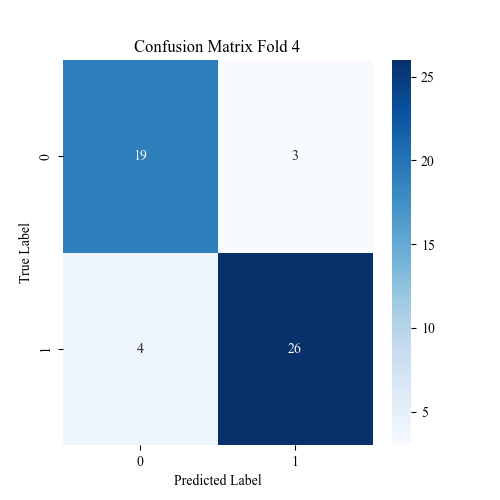

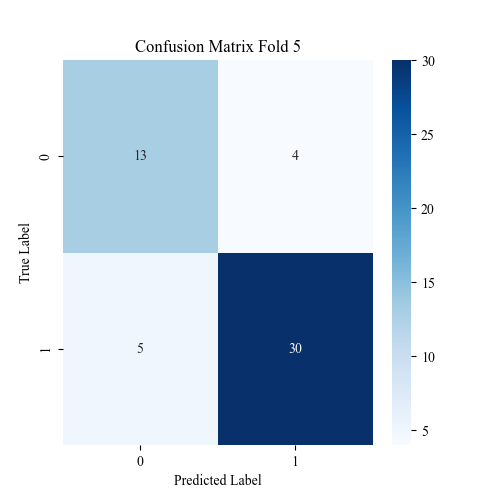
**

**Supplement Figure 2: Confusion matrix diagram of five-fold cross-validation of RFE-Voting**

**Supplementary Table 1. Detailed features for LASSO-BNB Model.**

| Features of Modeling |
| --- |
| Boundary |
| Grid |
| Wavelet_LH_glcm_MCC |
| Shape |
| Enhancement of behind echo |
| Sex |
| Homogeneity |
| Calcification |
| Wavelet_LL_glcm_InverseVariance |
| Wavelet_HH_glszm_SizeZoneNonUniformityNormalized |
| Wavelet_LL_glrlm_LongRunLowGrayLevelEmphasis |
| Original_ngtdm_Busyness |

**Supplementary Table 2. Detailed features for RFE-Voting Model.**

| Features of Modeling |
| --- |
| Sex |
| Age |
| Grid |
| Alder |
| Original_firstorder_Skewness |
| Wavelet_LH_firstorder_Skewness |
| Wavelet_LH_glcm_ClusterShade |
| Wavelet_LH_glcm_Correlation |
| Wavelet_LH_glcm_Imc2 |
| Wavelet_HH_glrlm_GrayLevelNonUniformityNormalized |
| Wavelet_LL_firstorder_Skewness |
| Wavelet_LL_gldm_DependenceVariance |
| Wavelet_LL_ngtdm_Busyness |
